# Morphological investigation and 3D simulation of plasmonic nanostructures to improve the efficiency of perovskite solar cells

**DOI:** 10.1038/s41598-023-46098-9

**Published:** 2023-10-30

**Authors:** Mohammad Hosein Mohammadi, Mehdi Eskandari, Davood Fathi

**Affiliations:** 1 Department of Electrical and Computer Engineering, Tarbiat Modares University (TMU), Tehran, Iran; 2 Nanomaterial Research Group, Academic Center for Education, Culture and Research (ACECR) on TMU, Tehran, Iran

**Keywords:** Solar cells, Solar cells, Solar energy and photovoltaic technology

## Abstract

The light absorption process is a key factor in improving the performance of perovskite solar cells (PSCs). Using arrays of metal nanostructures on semiconductors such as perovskite (CH_3_NH_3_PbI_3_), the amount of light absorption in these layers is significantly increased. Metal nanostructures have been considered for their ability to excite plasmons (collective oscillations of free electrons). Noble metal nanoparticles placed inside solar cells, by increasing the scattering of the incident light, effectively increase the optical absorption inside PSCs; this in turn increases the electric current generated in the photovoltaic device. In this work, by calculating the cross-sectional area of dispersion and absorption on gold (Au) nanoparticles, the effects of the position of nanoparticles in the active layer (AL) and their morphology on the increase of absorption within the PSC are investigated. The optimal position of the plasmonic nanoparticle was obtained in the middle of the AL using a three-dimensional simulation method. Then, three different morphologies of nano-sphere, nano-star and nano-cubes were investigated, where the short-circuit currents (J_sc_) for these three nanostructures were obtained equal to 19.01, 18.66 and 20.03 mA/cm^2^, respectively. In our study, the best morphology of the nanostructure according to the J_sc_ value was related to the nano-cube, in which the device power conversion efficiency was equal to 16.20%, which is about 15% better than the PSC with the planar architecture.

## Introduction

Today, due to the growing consumption of energy and the constraints that create non-renewable energy, human beings seek to replace renewable and clean resources in all areas. One of the new energies is solar energy, which can be used in heating sources or in a photovoltaic device^1–3^. Solar cells are photovoltaic devices that produce electrical energy by absorbing sunlight within the visible spectrum. The use of solar cells was introduced in the late nineteenth century. Initially, the production of electrical energy from sunlight was proposed for space applications, but with the development of silicon technology, the widespread use of this device was considered^4–7^. After silicon, the focus shifted to low-cost materials based on which solar cell production would be cost-effective, leading to the production of a new generation of solar cells^8–10^. Materials such as polymers and dye sensitization were used in the construction of solar cells, the cost of which was significantly reduced compared to the first generation^11–14^. Recently, perovskite materials for making solar cells have received a lot of attention. Properties such as high optical absorption coefficient, high mobility for carriers compared to polymeric materials and high diffusion length and band gap adjustment are among the electrical and optical properties considered in perovskite materials^15–18^. Perovskite solar cells (PSCs) are the third generation of solar cells that have shown many capabilities so far that they are considered as the main alternative to expensive mineral solar cells^19–22^. One of the challenges facing researchers in the field of solar cells is to achieve high efficiency while reducing manufacturing costs that the use of metal nanostructures has the potential to meet these two requirements. potential advancements in nanotechnology may open the door to the production of cheaper and slightly more efficient solar cells^23–25^. In^26^, a bottom-up bilateral modification strategy is proposed by incorporating arsenazo III (AA), a chromogenic agent for metal ions, to regulate SnO_2_ nanoparticles. AA can be complexed with uncoordinated Sn^4+^/Pb^2+^ in the form of multidentate chelation. (Rb_0.02_(FA_0.95_Cs_0.05_)_0.98_PbI_2.91_Br_0.03_Cl_0.06_) is boosted from 20.88 to 23.17% with a high open-circuit voltage (V_oc_) exceeding 1.18 V and ultralow energy losses down to 0.37 eV. In addition, the optimized devices also exhibit superior stability. In an experimental effort reported in^27^, the modulation of perovskite crystallization kinetics and halide ion migration through chlorobenzene (CB) antisolvent with bis(pentafluorophenyl)zinc (Zn(C_6_F_5_)_2_) additive has been explored. Furthermore, under 1-m-deep water, CsPbIBr_2_ PSCs display a PCE of 14.18%. These findings provide an understanding of the development of phase-segregation-free CsPbIBr_2_ films and showcase the prospective applications of CsPbIBr_2_ PSCs in underwater power systems. This study^28^ investigates the effects of introducing two functional urea-based molecules, biuret (BU) and dithiobiuret (DTBU), into the PbI_2_ precursor solution on the absorber layer and overall device performance. The highest achieved power conversion efficiency was 23.50%. After 1300 h of storage under unpackaged conditions at 30–40% humidity, the devices maintained 93% of their initial efficiency. Conversely, the devices prepared with DTBU doping exhibited inferior performance and stability, displaying power conversion efficiency below 10% and faster degradation under the same humidity conditions.

Plasmonic is an emerging field that makes use of the nano scale properties of metals. Though plasmonic is a wide area of study, its application for solar cells has seen a recent surge of interest^29,30^. Metals support surface plasmons that are the collective oscillation of excited free electrons and characterized by a resonant frequency^31^. They can be either localized as for metal nanoparticles or propagating as in the case of planar metal surfaces^32^. By manipulating the geometry of the metallic structures, the surface plasmon resonance (SPR) or plasmon propagating properties can be tuned depending on the applications^33^. The resonances of noble metals are mostly in the visible or infrared region of the electromagnetic spectrum, which is the range of interest for photovoltaic applications^34,35^. The surface plasmon resonance is affected by the size, shape and the dielectric properties of the surrounding medium. Plasmon is formed when a large number of moving electrons belonging to a metal nanoparticle are out of equilibrium. Due to the collision of electromagnetic radiation, free metal electrons oscillate at a certain plasma frequency relative to the positive ions. The vibration of this plasma is called plasmon, which, like phonons and photons (resulting from optical and mechanical vibrations, respectively)^36–39^. The oscillation frequency for Au nanoparticles occurs in the visible region and creates a strong absorption spectrum in this region. As the size and shape of the nanoparticles change, the peak of the plasmons changes. Because plasmons are derived from the classical-quantum of plasma oscillations, most of their properties are obtained directly from Maxwell's equations. A localized surface plasmon resonance (LSPR) is another type of plasmon that related to the collective vibration of electrons in small volumes, such as metal nanoparticles^40^. The condition for this phenomenon to occur is that the particle size is smaller than the electromagnetic wavelength of the collision with them. The electromagnetic wave, by moving the free electrons, creates an electric dipole on the surface of the particle, which induces the opposite charge on the adjacent particles. In fact, this phenomenon occurs when the frequency of the light entering the particle causes the mass oscillation of the electrons in the particle's conduction band. As a result, from the oscillation of the conducting electrons due to the collision with the electric field of electromagnetic radiation, a strong absorption peak is obtained at the same frequency^41,42^. According to the given explanations, it can be concluded that the use of metal nanoparticles to stimulate plasmon is an efficient method to increase the PCE of PSCs. Here are some of the things that have been done in this field. In 2011, Di Qu et al.^43^ used metal nanoparticles in the active layer (AL) of the PSC to determine the effect of nanoparticles on absorption. It was found that metal nanoparticles with a radius of 15 nm increased the absorption by about 10% in the AL. Focus in^44^ was directed towards a crucial aspect of nanostructures, which involves optimizing and effectively managing light to minimize light loss and achieve a high Power Conversion Efficiency (PCE) for Photovoltaic Solar Cells (PSCs). To achieve this goal, we strategically employed plasmonic Nanoparticles (NPs) at various positions within the electron layer transfer (ETL), active layer, and hole transfer layer (HTL). Our findings indicate that the optimal placement for achieving the highest PCE is at the top of the HTL. Additionally, we introduced an additional layer, serving as a complementary layer (CH_3_NH_3_SnI_3_), to further enhance efficiency. This resulted in notable values for key parameters, including a short-circuit current (J_sc_) of 27.81 mA/cm^2^, an open-circuit voltage (V_oc_) of 0.949 V, a fill factor (FF) of 81.77%, and an overall PCE of 21.85%. In^45^, it is provided a comprehensive quantitative examination of the absorption spectrum in a perovskite solar cell made of CH_3_NH_3_PbI_3_ using an array of nanoparticles (NPs). Findings revealed that the incorporation of an array of gold nanoparticles (Au Nano spheres) results in an average absorption enhancement of over 45%, in contrast to only 27.08% for the baseline structure lacking NPs. Additionally, this study explored the combined impact of this engineered absorption enhancement on the electrical and optical performance parameters of the solar cell using one-dimensional solar cell capacitance measurements. These measurements indicated a PCE of approximately 30.4%, a substantial improvement compared to the PCE of approximately 21% observed in cells lacking nanoparticles Also in ref.^46^, which is an experimental work, metal nanoparticles in the form of Au@SiO_2_ (core–shell) were used to increase the optical absorption. By changing the shell radius in the core–shell structure used, the J_sc_ from 10.45 (mA/cm^2^) to 11.58 (mA/cm^2^). In 2019, according to Report^47^, Au, Ag and Al composition were used in the form of metal nanoparticles and the results obtained in this report showed that the best absorption is related to nanoparticles with Ag composition. Therefore, it can be concluded that the use of nanoparticles helps to increase the absorption, but to maximize the absorption in the AL, some optimization must be done. Here we are going to first examine the position of the nanoparticles in the AL. To achieve a suitable absorption efficiency, we compare the morphology of nanoparticles in three structures, nano-sphere, nano-star and nano-cube. The present report is divided into 4 sections. The first part gives a brief description of PSCs and how plasmon function in this type of cell. Section "Theory and simulation details" describes the simulation process and then section "Results and discussion" describes the simulation results in detail. In the final part, we have stated the conclusion of the work done.

## Theory and simulation details

### Theory

To obtain the required specifications in a solar cell, we used two optical-electric models. We calculated the results of the optical and electrical models by finite-difference time-domain (FDTD) and finit element method (FEM) methods, respectively. In the fdtd method, we first obtain the Maxwell equations for the whole device. This means that the calculation of the electromagnetic field values progresses at discrete steps in time. however, the main reason for using the FDTD approach is the excellent scaling performance of the method as the problem size grows.

1 $$ \frac{{\partial {\text{H}}}}{{\partial {\text{t}}}} = \frac{ - 1}{\mu }\nabla \times E, $$

2 $$ \varepsilon \frac{\partial E}{{\partial t}} = - \nabla \times H - \sigma E, $$

where *E* is the electric field intensity, *H* is the magnetic field intensity, *ε* is the permittivity, *µ* is the permeability, and σ is the electric conductivity. After calculating the electric field, it is now possible to measure the amount of absorption at any point in the device using Eq. 3.

3 $$ P_{abs} = - 0.5\omega \left| E \right|^{2} imag\left( \varepsilon \right), $$

**Figure 1:**
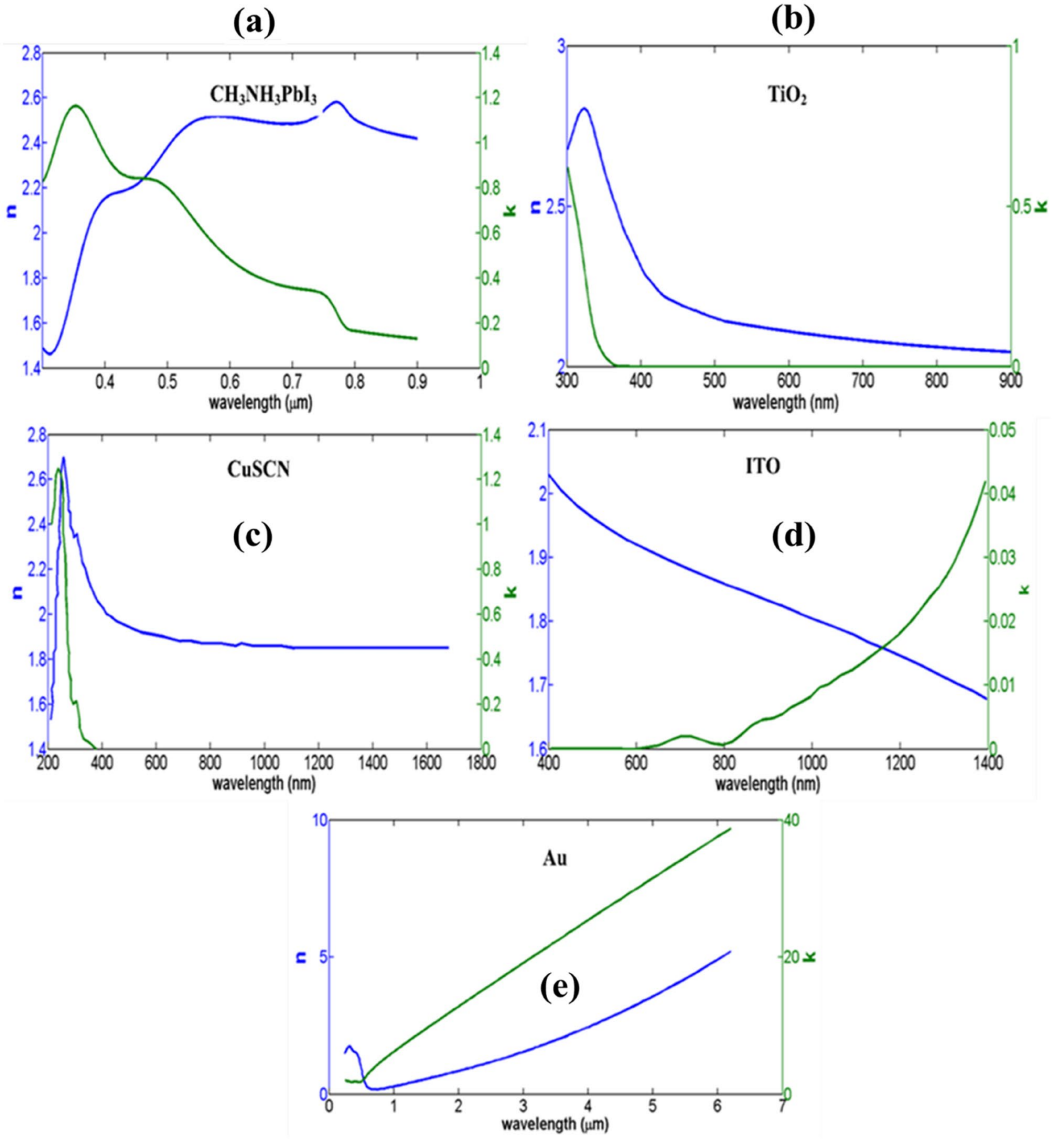
refractive index and extinction coefficient of (**a**) CH_3_NH_3_PbI_3_, (**b**) TiO_2_, (**c**) ITO, (**d**) CuSCN and (**e**) Au.

In this equation (Eq. 3), ${\left|E\right|}^{2}$ is the intensity of the electric field, which is obtained by solving Maxwell's equations, and on the other hand, by having the imaginary part the permittivity, Eq. 3 can be solved. Now using Eq. 4, the carrier generation rate can be obtained^48^.

4 $$ G_{OPT} = \frac{{\varepsilon^{\prime \prime } E^{2} }}{2\hbar }, $$

where *E* is the electric field intensity, *h* is the Plank’s constant, and $\varepsilon "$ is the imaginary part of the relative permittivity. Equation 4 is a key equation for calculating J_sc_. Optical properties for the materials used in the simulation are obtained from valid experimental work^49–53^. In the optical model, we also report refractive index n and extinction coefficient k of each layer in Fig. 1. Considering the carrier generation rate (G_opt_) and the recombinant mechanism (R_total_), which are radiant and non-radiant processes, respectively. Using Eq. (5), we obtained the optical current of the device.

5 $$ J_{sc} = {\text{q}}\left( {R_{total} - G_{opt} } \right), $$

where, q is the electron charge. Now we have used the FEM method to obtain the results of the electrical model, including the V_oc_. In this method, the equations of continuity and Poisson are solved.

6 $$ \nabla \cdot \left( {\varepsilon_{0} \cdot \varepsilon_{r} \nabla \varphi } \right) = - \rho , $$

7 $$ \frac{\partial n}{{\partial t}} = \frac{1}{q}\nabla j_{n} + G_{n} - U_{N} , $$

8 $$ \frac{\partial p}{{\partial t}} = \frac{1}{q}\nabla j_{p} + G_{p} - U_{p} , $$

where *q* is the charge of electron, *φ* is the electrostatic potential, *ε*_0_ is the vacuum permittivity and *U*_*P*_ are the rates of recombination of electrons and holes, respectively. *J*_*n*_ and *J*_*p*_ are the current densities of electrons and holes, respectively, *G*_*n*_ and *G*_*p*_ are the total generation rates of electrons and holes, respectively. In Eq. 8 and 9, the values of *G*_*n*_ and *G*_*p*_ are considered equal to *G*_*tot*_. We use Eq. 9 to draw the characteristic curve J-V.

9 $$ J\left( V \right) = J_{dark} - J_{sc} = J_{0} \left( {exp\left( {\frac{eV}{{nKT}}} \right) - 1} \right) - qG_{OPT} \left( {L_{n} + L_{p} } \right), $$

According to Eq. (9), ${J}_{dark}$ is the dark current of the device and when there is no carrier generation.

### Simulation details

**Figure 2:**
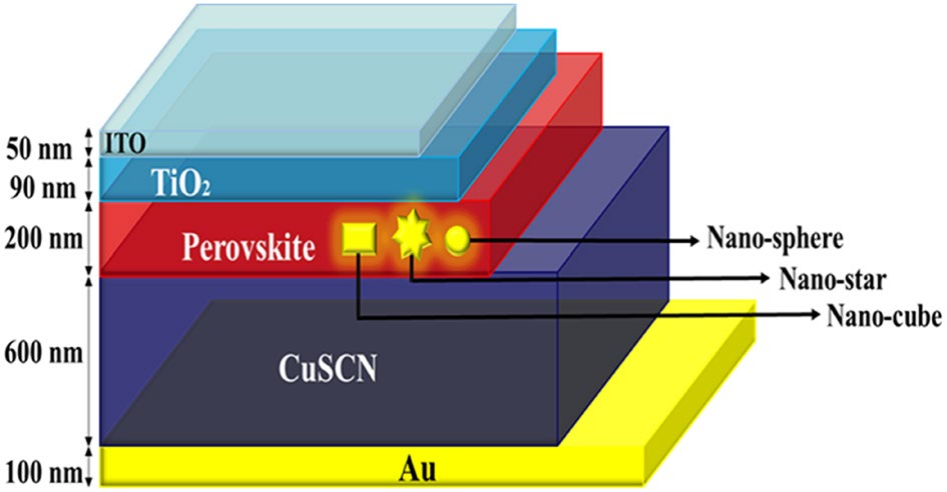
The schematic of the perovskite solar cell with an Au nanoparticle (NP).

Figure 2 demonstrate the PSC structure with plasmonic nanostructre (nano-sphere, nano-star and nano-cube) that has been simulated and studied. To obtain accurate and reliable results, we performed the simulation in 3D. TiO_2_ and CuSCN are two materials with type n and p are used as ETL and HTL respectively. Perovskite with the formula of chemistry CH_3_NH_3_PbI_3_ was also selected as the absorbent material. In addition, FTO and Au (φ_m_ = 5.1 eV) are selected as the front and back contact as well ideal ohmic and Schottky contacts with surface recombination speed of 1 × 10^7^ cm/s for front and back contacts were used respectively., respectively. The composition of plasmonic nanostructures is also considered to be Au metal. In the optical model, all the layers were selected, and unlike this model, in the electric model, only the ETL, perovskite and HTL are selected. In the electrical model, the carrier recombination at the interfaces between TiO_2_, perovskite and CuSCN is neglected. The trap-assisted recombination model is used to simulate the recombination rate within bulk materials, which is directly related to the volume of the layers. Utilizing the Trap-Assisted Recombination model to set the electron and hole recombination rates in band-gap semiconductors such as perovskite under low electric fields. This feature is an expanded version of the original Shockley–Read–Hall Recombination feature, with new options to allow for more detailed modelling of traps. This option corresponds to the original Shockley–Read–Hall model for steady state recombination via states located at the midgap. The Shockley–Read–Hall recombination rate is defined as^54^

10 $$ R_{n} = R_{P} = \frac{{np - n_{i,mod}^{2} }}{{\tau_{p} \left( {n + n_{1} } \right) + \tau_{n} \left( {p + p_{1} } \right)}}, $$

with

11 $$ n_{i,mod} = \gamma_{n} \gamma_{p} \sqrt {N_{c0} N_{v0} } e^{{\left( { - \frac{{E_{g} - \Delta E_{g} }}{{2V_{th} }}} \right)}} , $$

12 $$n_{1} = \gamma_{n} \sqrt {N_{c0} N_{v0} } e^{{\left( { - \frac{{E_{g} - \Delta E_{g} }}{{2V_{th} }}} \right)}} e^{{\left( {\frac{{\Delta E_{t} }}{{V_{th} }}} \right)}} , $$

13 $$ p_{1} = \gamma_{p} \gamma_{p} \sqrt {N_{c0} N_{v0} } e^{{\left( { - \frac{{E_{g} - \Delta E_{g} }}{{2V_{th} }}} \right)}} e^{{\left( {\frac{{\Delta E_{t} }}{{V_{th} }}} \right)}} , $$

where γn and γp are the electron and hole degeneracy factors, N_c,0 _and N_v,0 _are the effective densities of states for the conduction and valence band, Eg is the band gap and ΔEg the band gap narrowing energies (SI unit: V), scaled by the electron charge, q. V_th_ = k_B_T/q, where k_B_ is Boltzmann’s constant and T is the temperature. The parameters τ_n_ and τ_p_ are carrier lifetimes (SI unit: s) and E_t_ is the trap energy level (SI unit: V), scaled by the electron charge. The electron lifetime, SRH τ_n_ (SI unit: s) is taken from material and the hole lifetime, SRH τ_p_ (SI unit: s) is taken from material. In Table 1, ε_r_ denotes the relative permittivity N_c_ and N_v_ are effective conduction band and valance band densities, µ_n_ and µ_p_ are electron and hole mobilities, χ is electron affinity, E_g_ is the band gap, N_A_ and N_D_ are acceptor and donor densities, and τ_p_, τ_n_ are the life times of hole and electron^53,55–68^.

**Table 1:** Electrical parameters of the PSC.

Parameter	TiO_2_	CH_3_NH_3_PbI_3_	CuSCN
Relative permittivity (*ε*_r_)	9	6.5	10
Effective conduction band density (N_C_ (cm^−3^))	1 × 10^19^	1.66 × 10^19^	1.79 × 10^19^
Effective valance band density (N_V_ (cm^−3^))	1 × 10^19^	5.41 × 10^19^	2.51 × 10^19^
Electron/hole mobility (µ_n_/µ_p_ (cm^2^/V))	20/10	50/50	25/25
Electron affinity (χ (eV))	4	3.93	1.9
Band gap energy (E_g_ (eV))	3.2	1.55	3.4
Acceptor concentration (N_A_ (cm^−3^))	–	5 × 10^13^	5 × 1018
Donor concentration (N_D_ (cm^−3^))	5 × 10^18^	–	–
Electron/hole life time (τ_n_/τ_p_ (ns))	5/2	8/8	5/5

## Results and discussion

**Figure 3:**
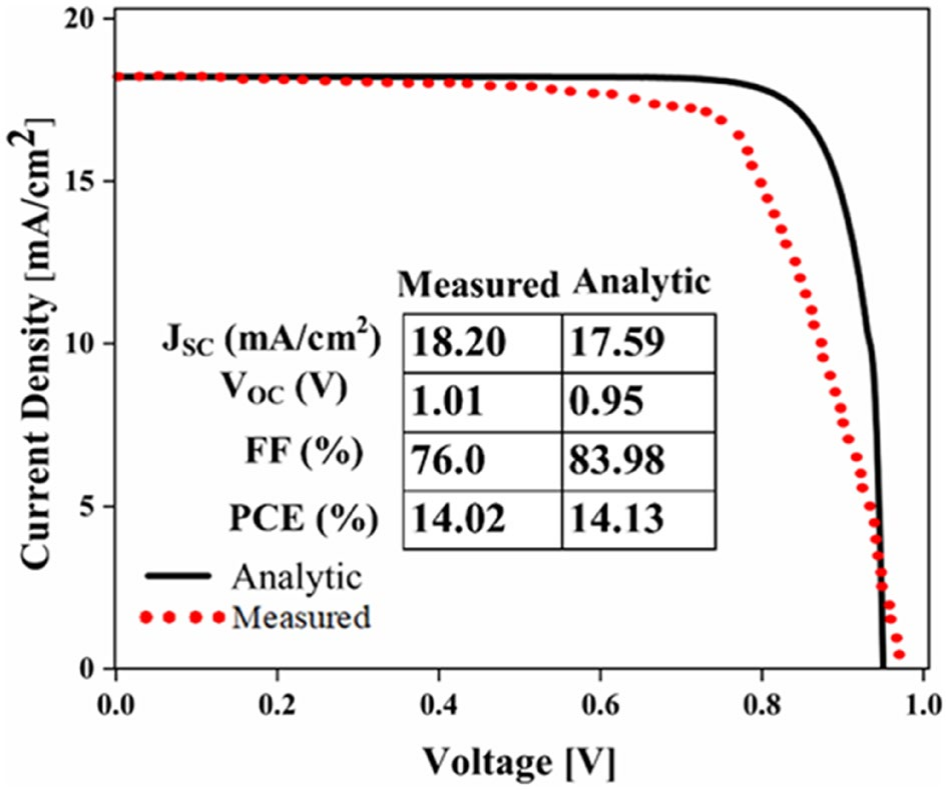
Validation of simulation results compared to analytic results.

**Figure 4:**
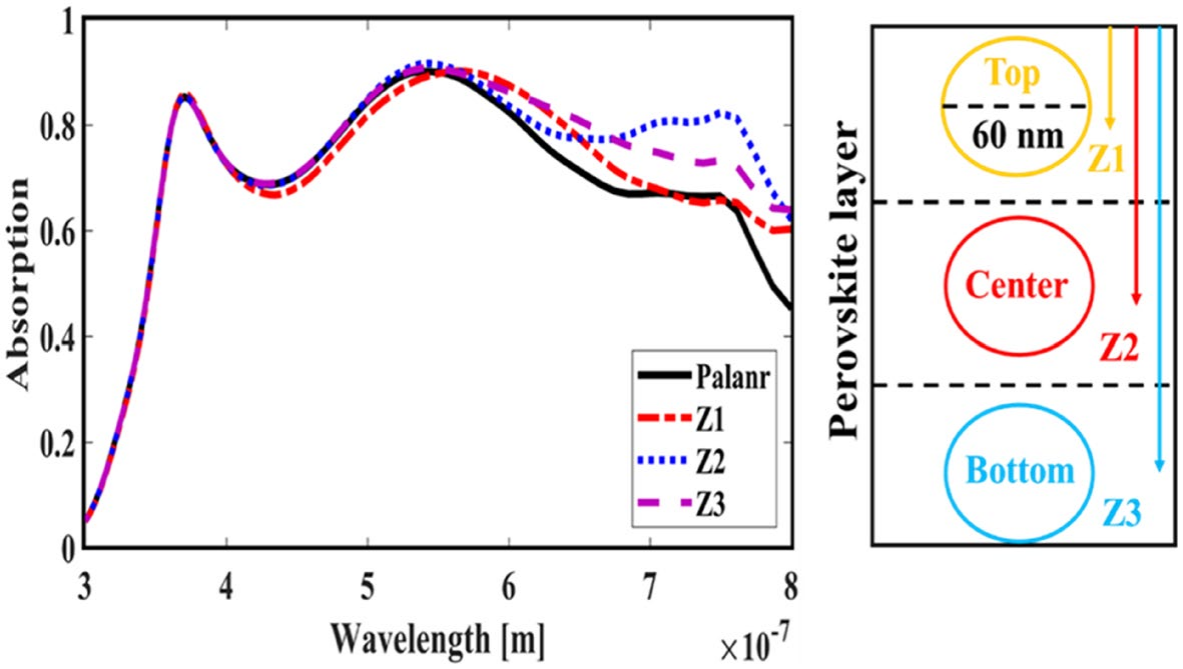
Characterization of the AL absorption spectrum in the presence of nano-spheres at three Z1, Z2 and Z3 positions.

**Figure 5:**
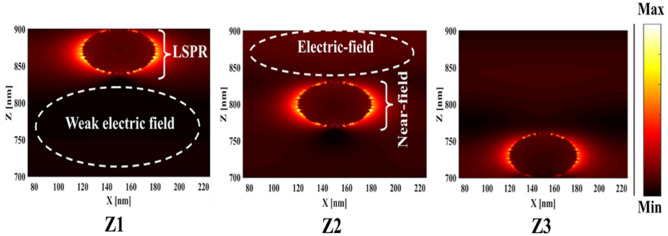
Electric field profiles for nano-spheres at three positions Z1, Z2 and Z3.

**Figure 6:**
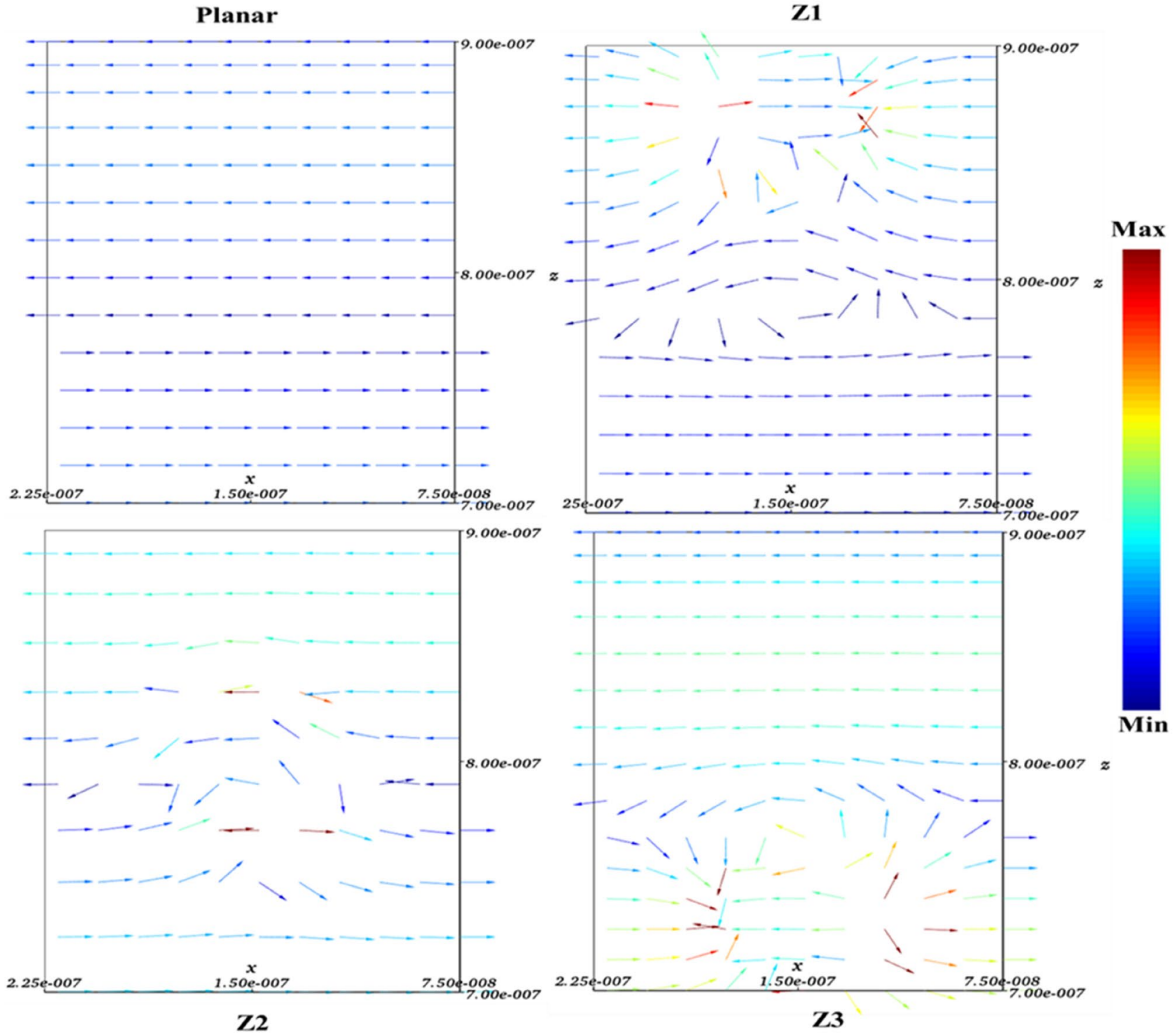
Variations of electric field vectors in terms of dimensions (x and z) of the active layer for nano-spheres in the planar Z1, Z2 and Z3 states.

**Figure 7:**
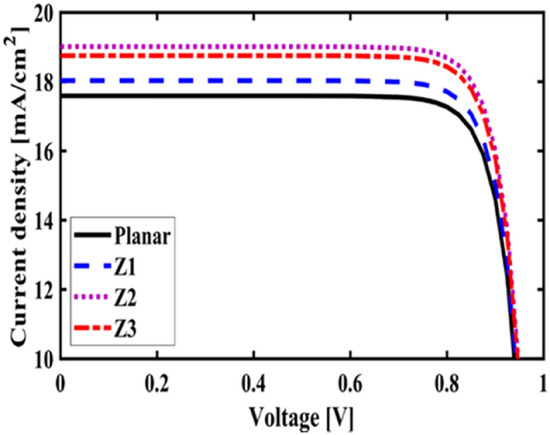
Comparison of the J–V characteristic proposed PSC for planar, Z1, Z2 and Z3 states.

**Figure 8:**
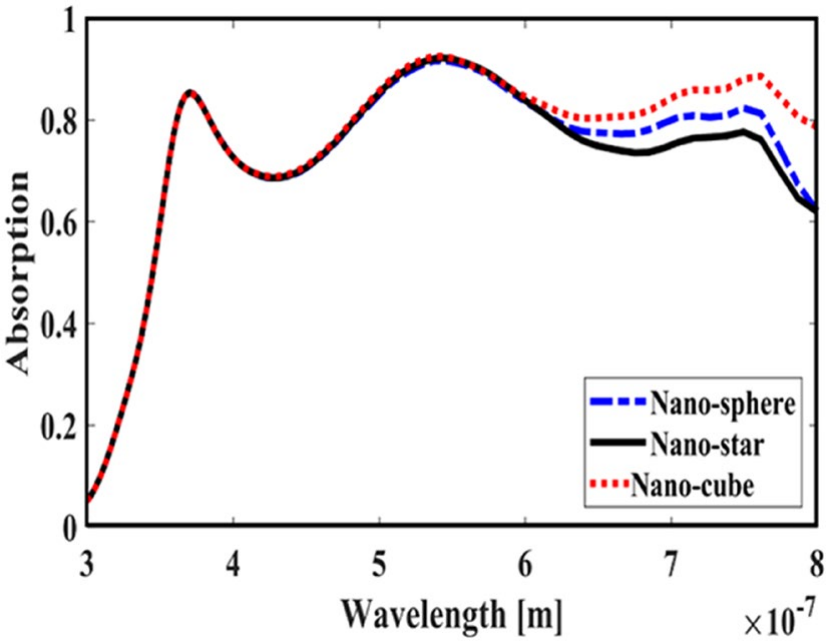
Active layer absorption spectrum in the presence of nano-spheres, nano-star and nano-cube.

**Figure 9:**
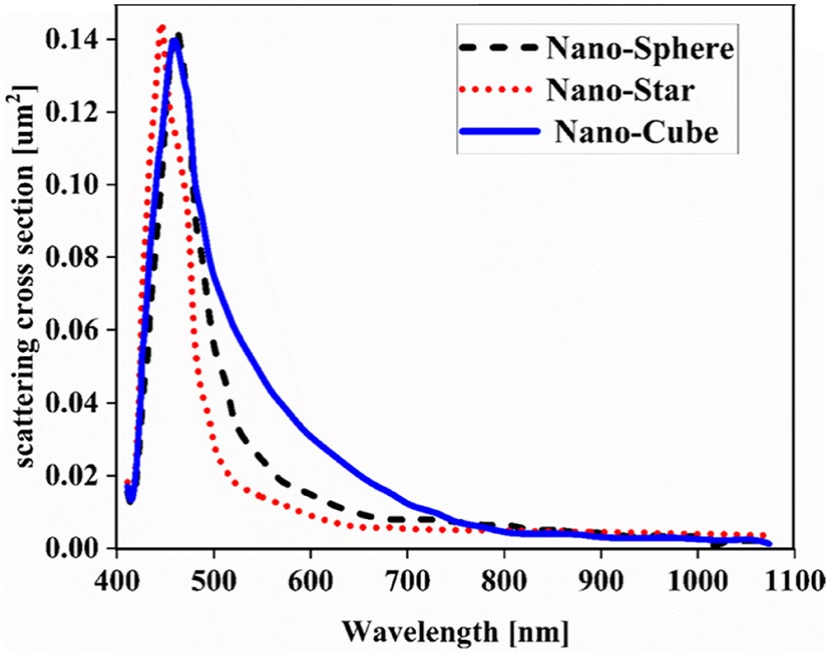
Simulated scattering cross section of Au nano sphere (dashed line), star (dotted line) and cube (solid line).

**Figure 10:**
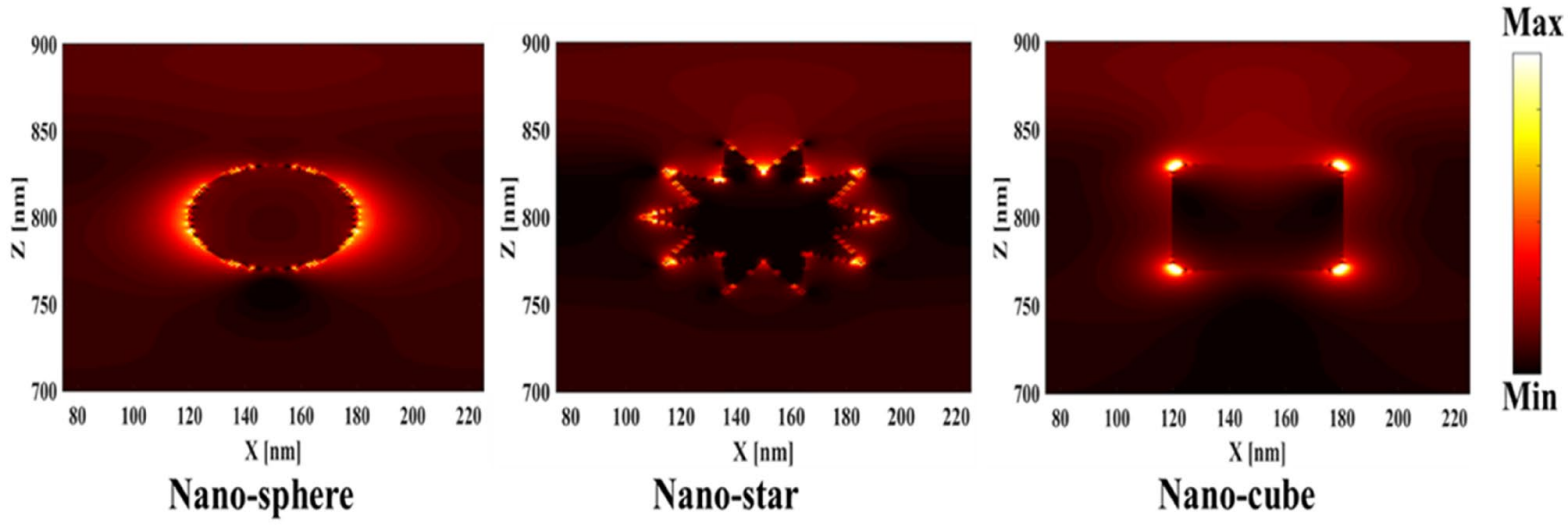
Comparison of electric field profiles for proposed PSC including nano-spheres, nano-stars and nano-cubes.

**Figure 11:**
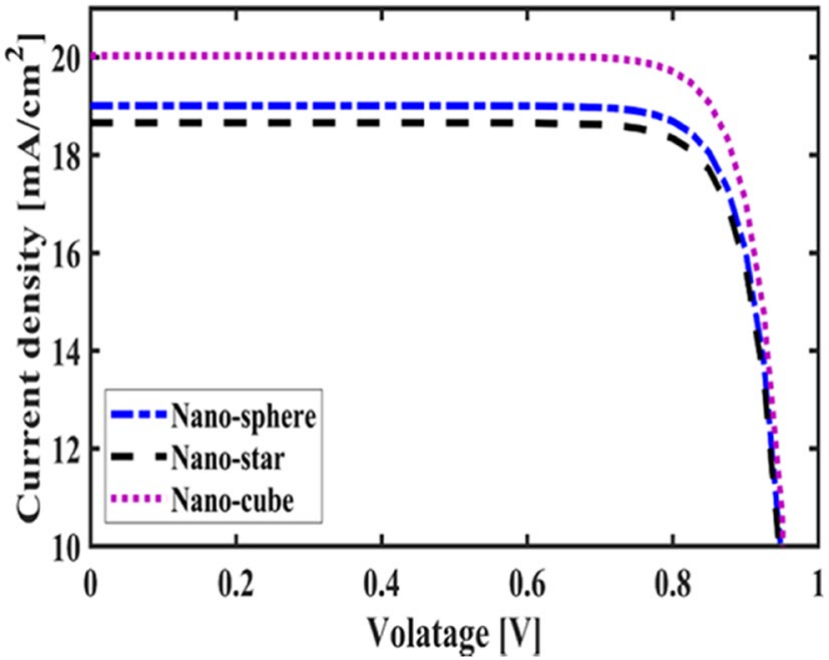
The J–V characteristic proposed PSC for three different morphology metal nanostructure such as nano-sphere, nano-star and nano-cube.

First, to prove the accuracy of the results we obtain, we compare the simulation results with an experimental work given in reference^69^. Figure 3 shows the validation of our results compared to the experimental work. As can be seen, both results are in a good balance so the accuracy of the information reported in this study can be relied upon. To begin, we placed the nano-sphere in the AL in three different positions (top, center and bottom) and obtained the results for this state. For this purpose, we obtained the absorption spectrum of the AL (perovskite) in the presence of nano-spheres in three different positions. Figure 4 illustrates this point. For convenience, we have shown the three positions top, center and bottom with the symbol Z1, Z2 and Z3 respectively. According to Fig. 4, when the nano-sphere is in the Z1 position at the wavelengths of 400 to 550 nm, the absorption spectrum is less than the planar spectrum. The reason for this phenomenon can be attributed to the reflection of light in the initial stage by nano-spheres. As the wavelength increases, plasmon of nano-spheres are formed, resulting in increase of the AL absorption. By placing the nano-spheres in the Z2 and Z3 positions, the absorption spectrum is greater at the total wavelength than the planar spectrum. However, when the nano-sphere is in the Z2 position, the AL reaches its maximum due to the stimulation of the nanosphere plasmon. To clarify the issue, we calculated the electric field profile of the AL for the nano-sphere at three Z1, Z2 and Z3 positions and showed it in Fig. 5. When the nano-sphere is in the Z1 position (Fig. 5, Z1), light interacts with the nano-sphere as soon as it enters the AL, and some of the light is scattered by the nanosphere. This causes the light to not reach the lower parts of the AL well, and as it turns out, there is a weak electric field in these areas (between 700 and 800 nm). In the next step, we placed the nano-sphere in the center of the AL (Z2) and it was found that the electric field was continuously distributed along the AL relative to the Z1-state. In this regard, the electric field generated by LSPR appears as a near-field around the nanosphere. At the end, we placed the nanosphere in the Z3 position, and the electric field in the AL is weaker than in the Z2 state. In order to explain these events more precisely, we have shown the electric field profile for the nanosphere in three Z1, Z2 and Z3 positions as a vector in Fig. 6. Figure 6 shows the electric field vectors in the perovskite layer. The x direction represents the transverse direction of the structure, the z direction represents the longitudinal direction of the structure, and the y direction is the direction of the third dimension on the plane, which cannot be displayed in two dimensions. Figure 6 can help us better understand how nano-spheres function in increasing AL absorption. This figure shows the electric field vectors in the AL and specifies the direction of change in the electric field. According to Fig. 6, when the nano-sphere is not present in the AL, the electric field vectors exhibit the same behaviour. In fact, this indicates that the electric field is uniform distribution without any changes in the AL. When we place the nano-sphere in the Z1 position of the AL, it is observed that the electric field vectors at the top of the AL have changed. However, in the lower part of the AL, the electric field vectors behave quite similarly to the planar state. The changes in the electric field in this state (Z1) are to be low and can be attributed to the greater reflection of light by the nanosphere. In Fig. 6 of the Z2 state, when the nano-sphere is in the Z2 position, the total electric field vectors change at all points of the AL. Part of this change is related to the formation of LSPR and the resulting near-fields. Another part of these changes is due to the scattering of light by the nano-sphere. This scattering is useful because the light scattered by the nano-sphere is reflected into the AL and leads to increasing the absorption of this layer. On the other hand, electric field vectors in the lower part of the AL are affected by the nano-sphere and lead to a change in the behaviour of electric field vectors in this region. When the nano-sphere is in the Z3 position, the electric field vectors are altered by LSPR stimulation around the nano-sphere, causing the electric field to accumulate and trap in this region. It is noteworthy at the top of the AL that the behaviour of electric field vectors with a planar state is exactly the same. But the intensity of electric field vectors is different from that of the planar state. This difference in intensity can be seen in the scattering of light by the nanosphere. After obtaining optical results, now it is time to obtain electrical results by FEM method. For this purpose, we have shown the J-V characteristics of the proposed PSC in the presence of nano-spheres in the planar, Z1, Z2 and Z3 states in Fig. 7. The J_sc_ for the planar, Z1, Z2 and Z3 is equal to 17.59, 18.03, 19.01 and 18.75 mA/cm^2^ respectively. As it turns out, the best current is for the nanosphere, which at position Z2 and its PCE was 15.33%. The other proposed PSC parameters are summarized in Table 2. Now that we have the best position (Z2) in the AL, to improve the absorption and more increase the efficiency, we focus on the morphology of nanoparticles and compare nanostructures such as nano-stars and nano-cubes. First, we obtained the absorption diagram of the AL in the presence of three nanostructures including nano-sphere, nano-star and nano-cube (Fig. 8). According to Fig. 8, the change of the absorption spectrum rate from the wavelength of 300 to 650 nm is almost constant. From the wavelength of 650 nm, changes in the absorption behavior of the AL appear. These changes can be known in the path of light reaching the nanostructures. As shown in Fig. 8, the nano-cube nanostructure generates the most absorption by LSPR stimulation in the AL. Of course, it is worth mentioning that according to the optimization we did in the position of nanostructures, we put three nanostructures in Z2 and then compared them. Figure 9, the scattering cross section of nano cube has higher values than sphere and star, especially in the range of λ > 500 nm of the solar spectrum. This means that the nano cube in structure is more efficient than sphere and star; because of the hybridization between the plasmonic modes of the cube and the perovskite. According to Fig. 8, It is shown that the absorption values of the geometrical structures are relatively small compared to scattering cross section values. nano cube is the structure that offers the highest scattering to the absorption ratio. According to the previous procedure, we obtained the electric field profile for three nanostructures and showed it in Fig. 10. Breaking points in a nanostructure can lead to condensation and accumulation of electric field. Hence, the breaking points in the nano-star trap the electric field, but the electric field is not completely accumulated around the nano-star. In fact, in this type of nanostructure, LSPR is not well formed compared to nanospheres. When we turned the nanostructure into a nano-cube, the electric field of the breaking points on the four sides of the nano-cube reached its maximum intensity, and the near-field is well formed. On the other hand, due to their special morphology, light scattering has occurred in the best possible way. Figure 11 shows the J-V characteristic proposed PSC for three different morphology metal nanostructure such as nano-sphere, nano-star and nano-cube. According to the optical characteristics and G_opt_, the highest J_sc_ belonged to nano-cube with a value of 20.03 mA/cm^2^, which has increased by 14% compared to the planar state. The electrical parameters for the nano-sphere and nano-star nano-cube embedded in the PSC AL are given in Table 3.

**Table 2:** Electrical parameter for proposed PSC in planar, Z1, Z2 and Z3 structure.

Structure	J_sc_ (mA/cm^2^)	V_oc_ (V)	FF (%)	PCE (%)
Planar	17.59	0.97	82.63	14.13
Z1	18.03	0.97	82.73	14.50
Z2	19.01	0.97	82.95	15.33
Z3	18.75	0.97	82.90	15.11

**Table 3:** Electrical parameter for proposed PSC in planar, nano-sphere, nano-star and nano-cube.

Structure	J_sc_ (mA/cm^2^)	V_oc_ (V)	FF (%)	PCE (%)
Planar	17.59	0.97	82.63	14.13
Nano-sphere	19.01	0.97	82.95	15.33
Nano-star	18.66	0.97	82.88	15.04
Nano-cube	20.03	0.97	83.16	16.20

## Conclusion

The LSPR amplification of metal nanoparticles create a strong near-electric field around the nanoparticle surface as well as a strong scattering of incident light. In order to use plasmonic resonance of nanoparticles, it is necessary to adjust the resonance. Plasmon resonance is a function of the composition, size and morphology of the nanoparticles as well as the refractive index of the medium. Therefore, by examining the scattering cross-section diagram and studying the maxima of this curve, the frequencies that have the highest scatter can be obtained, which are in fact the same resonant frequencies. First, we placed the gold nanoparticles in the AL of PSC structure in three different positions, namely the top, the middle and the bottom, and the best result was related to the nanoparticle that was in the middle. The J_sc_ value for this state was 19.01 mA/cm^2^. We further showed that for the three nanostructures nano-spheres, nano-stars, and nano-cubes, the absorption of the AL and the J_sc_ value were changed. According to this study, the best morphology due to having more breaking points was related to the nano-cube, in which case the J_sc_ value increased to 20.03 mA/cm^2^ and also the PCE to 16.20%. In summary, we have shown that the presence of metal nanoparticles in PSCs has a significant effect on increasing the optical absorption within the cell, and with increasing optical absorption, the number of electron–hole pairs generated increases, resulting in increase of the PCE of PSC.

## Data Availability

The datasets used and/or analyzed during the current study are available upon reasonable request from the corresponding author.
